# Failure Lifetime Evaluation Based on Accelerated Generalized Wiener Degradation Process Models with Random Diffusion Coefficients

**DOI:** 10.3390/e28050575

**Published:** 2026-05-21

**Authors:** Shanshan Li, Zaizai Yan

**Affiliations:** 1 College of Science, Inner Mongolia University of Technology, Hohhot 010051, China; lishanshan501@163.com; 2 College of Mathematics and Computer Science, Hetao College, Bayannur 015000, China

**Keywords:** generalized Wiener process, accelerated degradation model, diffusion coefficient, random effects, failure lifetime

## Abstract

This paper proposes a modeling framework for nonlinear degradation under constant-stress accelerated degradation testing (CSADT) to predict failure lifetime. The proposed employs a generalized Wiener process to characterize degradation, wherein the drift coefficient is stress-dependent and the heterogeneity in the diffusion coefficient is explicitly modeled. Random effects are introduced to capture volatility variability across degradation trajectories, and model parameters are estimated via the expectation–maximization (EM) algorithm. Using the law of total probability, the probability density function (PDF) and reliability function of failure lifetime under normal operating conditions are derived. The proposed model is validated using crack propagation simulation data and experimental wear scar width data from an alloy product. The results demonstrate that the proposed model improves prediction accuracy for failure lifetime and reliability, highlighting its potential utility in engineering applications.

## 1. Introduction

During product development, processes such as physics-of-failure analysis, performance testing, and inspection are routinely conducted, providing information related to product life and reliability characteristics. Effective utilization of this information provides a foundation for the reliability assessment of high-reliability, long-life products. For instance, Shi et al. [1] evaluated the reliability of complex multi-state systems. However, for many long-life products, degradation tests under normal operating conditions are both time-consuming and costly, making it difficult to observe significant degradation within a feasible timeframe. To address this challenge, accelerated degradation testing (ADT) employs elevated stress levels to expedite the degradation process. The degradation data collected under these high stress levels can then be used to predict the failure lifetime and reliability under normal stress conditions. Influenced by operating environments, applied stress levels, and internal material properties, the degradation process is often modeled as a stochastic process. Degradation is generally defined as the gradual deterioration of system performance over time. Such phenomena are prevalent in various metallic components and are particularly pronounced in mechanical and structural systems [2]. Commonly used stochastic processes models include the Wiener, Gamma, and inverse Gaussian processes [3,4,5]. Among them, the Wiener process, characterized by robust mathematical properties and clear physical interpretation, is widely applied to model non-monotonic degradation [6]. In linear scenarios, a Wiener process with linear drift is typically employed to model degradation [7,8]. For nonlinear cases, Whitmore et al. [9] and Ye et al. [10] utilized time-scale or logarithmic transformations to process degradation data [11,12]. However, not all nonlinear degradation processes can be linearized through such transformations, thereby limiting their applicability. Si et al. [13] proposed a nonlinear Wiener process and derived approximate probability density functions (PDFs) for the lifetime and remaining useful life (RUL) under the first passage time (FPT) conditions. Wang et al. [6,14] further developed a generalized Wiener process that simultaneously accounts for both nonlinear characteristics and temporal uncertainty, with the latter captured by non-standard Brownian motion. This generalized Wiener process model can describe degradation with arbitrary functional forms for the mean and variance. Furthermore, many commonly used models are special cases of this framework, demonstrating its broad applicability.

Accelerated degradation testing (ADT) is a widely adopted and effective method for the reliability assessment of high-reliability and long-life products [15]. Based on the stress application method, ADT is primarily categorized into constant-stress ADT (CSADT) and step-stress ADT (SSADT) [16]. Meeker and Escobar [17] provide a comprehensive overview of ADT methodologies. In CSADT, test specimens are divided into several groups and tested under different constant-stress levels, each held constant throughout the test duration. Due to its simple stress loading scheme and ease of implementation, CSADT has become a prominent approach in ADT [18], attracting considerable research interest. Chen et al. [19] optimized ADT designs within the CSADT framework by considering the impact of stress levels, random effects, and measurement errors. Duan et al. [20] proposed a nonlinear Wiener process model based on time-scale transformations for degradation modeling under CSADT, incorporating mixed random effects and measurement errors. Their approach was validated using stress relaxation data. Tang et al. [21] constructed a nonlinear degradation model by incorporating random effects within the CSADT framework. Liu et al. [22] explored a CSADT model based on generalized Wiener processes, comprehensively considering drift rate heterogeneity and the influence of multiple stresses. Li et al. [23] developed a multi-stress CSADT model with random effects and employed the EM algorithm for parameter estimation. In summary, across both single- and multi-stress scenarios, these models assume that drift coefficients depend on the accelerating stresses via established acceleration relationships, while other parameters remain stress-independent.

Due to variations in raw material quality, operating environments, and manufacturing processes, individual units of the same product may exhibit significant differences in degradation rates and volatility levels. This phenomenon is referred to as unit-to-unit heterogeneity [24,25,26]. To characterize this heterogeneity, random effects are incorporated into degradation models, where parameter variations are assumed to follow specific distributions. Various assumptions regarding model parameters and accelerated stresses have been proposed in Wiener-process-based accelerated degradation modeling. Zhao et al. [27] and Hao et al. [28] assumed that the drift coefficient depends on the accelerated stress levels. Cai et al. [29] treated the drift coefficient as a normally distributed random effect, established a nonlinear Wiener process model for accelerated degradation, derived the probability density function (PDF) of the remaining useful life (RUL), and employed Bayesian methods alongside the EM algorithm for parameter estimation. Li et al. [30] analyzed the impact of random effects in CSADT based on a generalized Wiener process by incorporating the principle of a constant acceleration factor. Whitmore et al. [9] and Liao et al. [31] proposed that both drift and diffusion coefficients are linked to accelerated stress levels. Wang et al. [32,33] utilized the constant acceleration factor principle to demonstrate that both parameters are governed by accelerated stress levels. Despite these advances, most existing studies that account for individual heterogeneity focus only on heterogeneity in the drift coefficient or degradation rate while assuming constant volatility, thereby neglecting possible heterogeneity in the diffusion coefficient. Ye et al. [34] investigated a model where heterogeneity in the drift coefficient is negligible and only the diffusion coefficient exhibits heterogeneity. Liang et al. [35] proposed a two-stage stochastic fluctuation inverse Gaussian degradation model and validated its effectiveness using train wheel degradation data. However, such models have received limited attention in the literature, necessitating further investigation.

The generalized Wiener process is widely applied in degradation modeling, because it encompasses many commonly used models [6]. Its flexibility in describing random degradation processes makes it particularly suitable for CSADT. Under the CSADT framework, individual products may exhibit significant trajectory variability due to differences in material grades or operating conditions, ranging from stable to highly volatile. Therefore, it is essential to account for unit-to-unit variability. In certain degradation scenarios, heterogeneity in the diffusion coefficient is even more pronounced than in the drift coefficient. In this study, a degradation model is developed based on the generalized Wiener process, in which a functional relationship between the drift coefficient and the accelerated stress level is incorporated, heterogeneity in the diffusion coefficient is explicitly accounted for, and the EM algorithm is employed for parameter estimation.

The structure of this paper is organized as follows. Section 2 proposes a generalized Wiener process model for CSADT and derives the PDF and reliability function of the failure lifetime under normal operating conditions. Section 3 presents parameter estimation via the expectation–maximization (EM) algorithm. Section 4 performs numerical simulations and case studies to validate the proposed model. Section 5 concludes the paper and suggests possible extensions for future work.

## 2. Constant-Stress Accelerated Degradation Model

### 2.1. Generalized Wiener Process

Let $X\left(t\right)$ denote the cumulative degradation amount of product performance at time t. The degradation process is modeled by a generalized Wiener process [6]

(1) $$X\left(t\right)=X\left(0\right)+\mu \Lambda \left(t;\theta \right)+\sigma_{B}B\left(\tau \left(t;\gamma \right)\right)$$

where $X\left(0\right)$ represents the initial degradation amount. For analytical convenience, assume $X\left(0\right)=0$. $\Lambda \left(t;\theta \right)$ and $\tau \left(t;\gamma \right)$ are strictly increasing functions of time t. θ and γ are parameters of the time-scale function, μ denotes the drift coefficient (i.e., the degradation rate), $\sigma_{B}$ denotes the diffusion coefficient, and $B\left(\tau \left(t;\gamma \right)\right)$ represents nonlinear Brownian motion, which describes the uncertainty in the degradation amount over time.

**Lemma 1** 
([6])**.** *Let* D *denote the failure threshold, and the time at which the product degradation amount* $X\left(t\right)$ *first reaches the threshold* D *is denoted by* T*,* $T=\inf\{t:X\left(t\right)\geq D\}$*. Based on the concept of the FPT, given* $\delta =\frac{1}{\sigma_{B}^{2}}$*, the approximate conditional PDF of the failure lifetime is expressed as*

$$f_{1}\left(t|\delta \right)≃\frac{1}{A_{1}}g_{1}\left(t|\delta \right),$$

(2) $$g_{1}\left(t|\delta \right)≃\frac{\delta^{\frac{1}{2}}}{\tau \left(t;\gamma \right)\sqrt{2\pi \tau \left(t;\gamma \right)}}\frac{d\tau \left(t;\gamma \right)}{dt}\exp\left[-\delta \frac{{\left(D-\mu \Lambda \left(t;\theta \right)\right)}^{2}}{2\tau \left(t;\gamma \right)}\right]\;\left[D-\mu \left(\Lambda \left(t;\theta \right)-\tau \left(t;\gamma \right)h\left(\tau \left(t;\gamma \right);\theta \right)\right)\right]$$

*where* $A_{1}=\int_{0}^{\infty }g_{1}\left(t|\delta \right)dt$, $h\left(\tau \left(t;\gamma \right);\theta \right)=\frac{d\Lambda \left(\tau^{-1}\left(s;\gamma \right);\theta \right)}{ds}{|}_{s=\tau \left(t;\gamma \right)}$.

### 2.2. Generalized Wiener Process Model with Accelerated Stress and Random Diffusion Coefficient

Assume that there are m accelerated stress levels denoted by $S_{0}\leq S_{1}<S_{2}<\cdots <S_{m}$, where $S_{0}$ represents the normal stress level and $S_{m}$ denotes the maximum stress level. The drift coefficient $\mu_{i}$ represents the degradation rate corresponding to the accelerated stress level $S_{i}$. The functional relationship between the drift coefficient and the accelerated stress levels is defined as $\mu_{i}=a\exp\left(bs_{i}\right)$ [36]. For the power-law acceleration model, the accelerated stress level is normalized as $s_{i}=\frac{\ln S_{i}-\ln S_{0}}{\ln S_{m}-\ln S_{0}}$. For the Arrhenius model, it is normalized as $s_{i}=\frac{1/S_{i}-1/S_{0}}{1/S_{m}-1/S_{0}}$, whereas for the exponential model, it is normalized as $s_{i}=\frac{S_{i}-S_{0}}{S_{m}-S_{0}}$. Considering a generalized Wiener process model that incorporates accelerated stress levels and random effects, the proposed model is denoted as $M_{1}$:

(3) $$X_{i}(t)=a\exp\left(bs_{i}\right)\Lambda \left(t;\theta \right)+\sigma_{B}B\left(\tau \left(t;\gamma \right)\right),\;i=1,2,\cdots ,m,$$

where the diffusion coefficient $\delta =\frac{1}{\sigma_{B}^{2}}$ is assumed to follow a special gamma distribution with shape parameter ν/2 and scale parameter ν/2, and the PDF of δ is given by $\pi \left(\delta \right)=\frac{{\left(\frac{\nu }{2}\right)}^{\frac{\nu }{2}}}{\Gamma \left(\frac{\nu }{2}\right)}\delta^{\frac{\nu }{2}-1}\exp\left(-\frac{\nu }{2}\delta \right),\nu ,\delta >0$. Where $\Gamma \left(\cdot \right)$ is the gamma function. To derive the PDF of the failure lifetime accounting for the random variable $\delta ∼Ga\left(\frac{\nu }{2},\frac{\nu }{2}\right)$, the following lemma is introduced.

**Lemma 2** 
([20])**.** *If* $Z∼Ga\left(\alpha_{z},\beta_{z}\right)$*,* w,m∈R*, then*

(4) EZZmexp−wZ=βZΓαZm+αZΓαZβZ+wm+αZ.

**Proof.**  Since $Z∼Ga\left(\alpha_{z},\beta_{z}\right)$,

EZZmexp−wZ=βZαZΓαZ∫0+∞Zm+αZ−1exp−w+βZZdZ=βZΓαZm+αZΓαZβZ+wm+αZ.

□

**Theorem 1.** 
*When the random variable* $\delta ∼Ga\left(\frac{\nu }{2},\frac{\nu }{2}\right)$*, the PDF of product’s failure lifetime at the normalized stress level* $s_{i}$ *is approximately expressed as*

$$f_{1}\left(t|s_{i}\right)≃\frac{1}{A_{M1}}g_{1}\left(t|s_{i}\right),$$

(5) $$g_{1}\left(t|s_{i}\right)≃\frac{\left[D-a\exp\left(bs_{i}\right)\left(\Lambda \left(t;\theta \right)-\tau \left(t;\gamma \right)h\left(\tau \left(t;\gamma \right);\theta \right)\right)\right]}{\tau \left(t;\gamma \right)\sqrt{2\pi \tau \left(t;\gamma \right)}}\frac{d\tau \left(t;\gamma \right)}{dt}\;\frac{{\left(\frac{\nu }{2}\right)}^{\frac{\nu }{2}}\Gamma \left(\frac{\nu +1}{2}\right)}{\Gamma \left(\frac{\nu }{2}\right){\left[\frac{{\left(D-a\exp\left(bs_{i}\right)\Lambda \left(t;\theta \right)\right)}^{2}}{2\tau \left(t;\gamma \right)}+\frac{\nu }{2}\right]}^{\frac{\nu +1}{2}}},$$

*where* $A_{M1}=\int_{0}^{\infty }g_{1}\left(t|s_{i}\right)dt$*,* $h\left(\tau \left(t;\gamma \right);\theta \right)=\frac{d\Lambda \left(\tau^{-1}\left(u;\gamma \right);\theta \right)}{du}{|}_{u=\tau \left(t;\gamma \right)}$*. The reliability function is given by*

(6) $$R_{1}\left(t|S_{i}\right)=1-\int_{0}^{t}f_{1}\left(u|s_{i}\right)du.$$

**Proof.**  For $\delta ∼Ga\left(\frac{v}{2},\frac{v}{2}\right)$, it follows from the total probability formula that $g_{1}\left(t|s_{i}\right)=\int_{-\infty }^{+\infty }g_{1}\left(t|\delta ,s_{i}\right)\pi (\delta )d\delta$, where π(δ) is the PDF of δ.

$$g_{1}\left(t|\delta ,s_{i}\right)≃\frac{\delta^{\frac{1}{2}}}{\tau \left(t;\gamma \right)\sqrt{2\pi \tau \left(t;\gamma \right)}}\frac{d\tau \left(t;\gamma \right)}{dt}\exp\left[-\delta \frac{{\left(D-a\exp\left(bs_{i}\right)\Lambda \left(t;\theta \right)\right)}^{2}}{2\tau \left(t;\gamma \right)}\right]$$

$$\left[D-a\exp\left(bs_{i}\right)\left(\Lambda \left(t;\theta \right)-\tau \left(t;\gamma \right)h\left(\tau \left(t;\gamma \right);\theta \right)\right)\right],$$

$$g_{1}\left(t|s_{i}\right)≃\frac{\left[D-a\exp\left(bs_{i}\right)\left(\Lambda \left(t;\theta \right)-\tau \left(t;\gamma \right)h\left(\tau \left(t;\gamma \right);\theta \right)\right)\right]}{\tau \left(t;\gamma \right)\sqrt{2\pi \tau \left(t;\gamma \right)}}\frac{d\tau \left(t;\gamma \right)}{dt}$$

$$E\left\{\delta^{\frac{1}{2}}\exp\left[-\delta \frac{{\left(D-a\exp\left(bs_{i}\right)\Lambda \left(t;\theta \right)\right)}^{2}}{2\tau \left(t;\gamma \right)}\right]\right\},$$

Given $m=\frac{1}{2}$ and $w=\frac{{\left(D-a\exp\left(bs_{i}\right)\Lambda \left(t;\theta \right)\right)}^{2}}{2\tau \left(t;\gamma \right)}$,

$$g_{1}\left(t|s_{i}\right)≃\frac{\left[D-a\exp\left(bs_{i}\right)\left(\Lambda \left(t;\theta \right)-\tau \left(t;\gamma \right)h\left(\tau \left(t;\gamma \right);\theta \right)\right)\right]}{\tau \left(t;\gamma \right)\sqrt{2\pi \tau \left(t;\gamma \right)}}\frac{d\tau \left(t;\gamma \right)}{dt}$$

$$\frac{{\left(\frac{\nu }{2}\right)}^{\frac{\nu }{2}}\Gamma \left(\frac{\nu +1}{2}\right)}{\Gamma \left(\frac{\nu }{2}\right){\left[\frac{{\left(D-a\exp\left(bs_{i}\right)\Lambda \left(t;\theta \right)\right)}^{2}}{2\tau \left(t;\gamma \right)}+\frac{\nu }{2}\right]}^{\frac{\nu +1}{2}}}$$

is obtained by Lemma 2. □

## 3. Statistical Inference

### 3.1. Likelihood Function for Degradation Data

In a CSADT, assume that the measurement time of the $k^{th}$ observation for the $j^{th}$ sample at the $s_{i}$ standardized accelerated stress level is recorded as $t_{i,1}^{j},t_{i,2}^{j},\cdots ,t_{i,p_{ij}}^{j}$, and the corresponding degradation measurements are denoted by $x_{i,j}=\left\{X\left(t_{i,1}^{j}\right),X\left(t_{i,2}^{j}\right),\cdots ,X\left(t_{i,p_{ij}}^{j}\right)\right\}$, and the increments between the $k^{th}$ and ${\left(k-1\right)}^{th}$ measurements are denoted by $\Delta x_{i,k}^{j}=X\left(t_{i,k}^{j}\right)-X\left(t_{i,k-1}^{j}\right)$, $\Delta x_{i,j}=\left\{\Delta x_{i,1}^{j},\Delta x_{i,2}^{j},\cdots ,\Delta x_{i,p_{ij}}^{j}\right\}$, $\Delta x_{i,k}^{j}∼N\left(a\exp\left(bs_{i}\right)\Delta \Lambda \left(t_{i,k}^{j},\theta \right),\frac{\Delta \tau \left(t_{i,k}^{j},\gamma \right)}{\delta_{j}}\right)$, i=1,2,⋯,m,$j=1,2,\cdots ,n_{i}$, $k=1,2,\cdots ,p_{ij}$, $\Delta \Lambda \left(t_{i,k}^{j},\theta \right)=\Lambda \left(t_{i,k}^{j},\theta \right)-\Lambda \left(t_{i,(k-1)}^{j},\theta \right)$, $\Delta \tau \left(t_{i,k}^{j},\gamma \right)=\tau \left(t_{i,k}^{j},\gamma \right)-\tau \left(t_{i,(k-1)}^{j},\gamma \right)$, $\Delta \Lambda_{i,j}=\left(\Lambda \left(t_{i,1}^{j},\theta \right),\Lambda \left(t_{i,2}^{j},\theta \right),\cdots ,\Lambda \left(t_{i,p_{ij}}^{j},\theta \right)\right)$.

When $\Lambda \left(t\right)=t^{\theta }$, $\tau \left(t\right)=t^{\gamma }$, at stress level $s_{i}$, given $\delta_{j}$, the PDF of the degradation increment for the $j^{th}$ sample under stress level $s_{i}$ is given by

(7) $$f\left(\Delta x_{i,j}|\delta_{j}\right)=\prod_{k=1}^{p_{ij}}\frac{1}{\sqrt{2\pi \Delta \tau \left(t_{i,k}^{j},\gamma \right)}}\delta_{j}^{\frac{1}{2}}\exp\left[-\frac{\delta_{j}{\left(\Delta x_{i,k}^{j}-a\exp\left(bs_{i}\right)\Delta \Lambda \left(t_{i,k}^{j},\theta \right)\right)}^{2}}{2\Delta \tau \left(t_{i,k}^{j},\gamma \right)}\right].$$

Since $\delta_{j}∼Ga\left(\frac{\nu }{2},\frac{\nu }{2}\right)$, the likelihood function for the $j^{th}$ sample under stress level $s_{i}$ can be expressed as

$$L_{i,j}\left(\Theta \right)=\int_{0}^{+\infty }\prod_{k=1}^{p_{ij}}\frac{1}{\sqrt{2\pi \Delta \tau \left(t_{i,k}^{j},\gamma \right)}}\delta_{j}^{\frac{1}{2}}\exp\left[-\frac{\delta_{j}{\left(\Delta x_{i,k}^{j}-a\exp\left(bs_{i}\right)\Delta \Lambda \left(t_{i,k}^{j},\theta \right)\right)}^{2}}{2\Delta \tau \left(t_{i,k}^{j},\gamma \right)}\right]\frac{{\left(\frac{\nu }{2}\right)}^{\frac{\nu }{2}}}{\Gamma \left(\frac{\nu }{2}\right)}{\delta_{j}}^{\frac{\nu }{2}-1}e^{-\frac{\nu }{2}\delta_{j}}d\delta_{j}$$

$$=\frac{{\left(\frac{\nu }{2}\right)}^{\frac{\nu }{2}}\Gamma \left(\frac{\nu +p_{ij}}{2}\right)}{\Gamma \left(\frac{\nu }{2}\right){\left(2\pi \right)}^{\frac{p_{ij}}{2}}\prod_{k=1}^{p_{ij}}\Delta \tau {\left(t_{i,k}^{j},\gamma \right)}^{\frac{1}{2}}{\left(\frac{\nu }{2}+\frac{1}{2}\sum_{k=1}^{p_{ij}}\frac{{\left(\Delta x_{i,k}^{j}-a\exp\left(bs_{i}\right)\Delta \Lambda \left(t_{i,k}^{j},\theta \right)\right)}^{2}}{\Delta \tau \left(t_{i,k}^{j},\gamma \right)}\right)}^{\frac{\nu +p_{ij}}{2}}}$$

(8) $$=\frac{\Gamma \left(\frac{\nu +p_{ij}}{2}\right)}{\Gamma \left(\frac{\nu }{2}\right){\left(\nu \pi \right)}^{\frac{p_{ij}}{2}}}\prod_{k=1}^{p_{ij}}\Delta \tau {\left(t_{i,k}^{j},\gamma \right)}^{\frac{1}{2}}{\left(1+\sum_{k=1}^{p_{ij}}\frac{{\left(\Delta x_{i,k}^{j}-a\exp\left(bs_{i}\right)\Delta \Lambda \left(t_{i,k}^{j},\theta \right)\right)}^{2}}{\Delta \tau \left(t_{i,k}^{j},\gamma \right)\nu }\right)}^{-\frac{\nu +p_{ij}}{2}}.$$

Let $\Omega_{i,j}=diag\left(\Delta \tau \left(t_{i,1}^{j},\gamma \right),\Delta \tau \left(t_{i,2}^{j},\gamma \right),\cdots ,\Delta \tau \left(t_{i,p_{ij}}^{j},\gamma \right)\right)$, product degradation amount $\Delta x_{i,j}∼T\left(ae^{bs_{i}}\Delta \Lambda_{i,j},\Omega_{i,j},\nu \right)$, where $T\left(\cdot ,\cdot ,\nu \right)$ denotes the $p_{ij}$-dimensional Student’s t distribution with ν degrees of freedom, and when ν→∞, Student’s t distribution approximates a multivariate normal distribution. Compared to the Wiener process, Student’s t-process demonstrates superior robustness, making it more effective for handling small-sample datasets with heavy-tailed characteristics. In this context, the degrees of freedom ν serve as a parameter to tune the model’s robustness.

The log-likelihood function for the degradation data is denoted by

(9) $$\ln L\left(\Theta \right)=\sum_{i=1}^{m}\sum_{j=1}^{n_{i}}\left\{\ln\Gamma \left(\frac{\nu +p_{ij}}{2}\right)-\ln\Gamma \left(\frac{\nu }{2}\right)-\frac{p_{ij}}{2}\ln\left(\nu \pi \right)-\frac{1}{2}\sum_{k=1}^{p_{ij}}\ln\left(\Delta \tau \left(t_{i,k}^{j},\gamma \right)\right)\right.\;\left.-\frac{\nu +p_{ij}}{2}\ln\left[1+\sum_{k=1}^{p_{ij}}\frac{{\left(\Delta x_{i,k}^{j}-a\exp\left(bs_{i}\right)\Delta \Lambda \left(t_{i,k}^{j},\theta \right)\right)}^{2}}{\Delta \tau \left(t_{i,k}^{j},\gamma \right)\nu }\right]\right\}.$$

As shown in Equation (9), it is computationally difficult to directly obtain parameter estimates using maximum likelihood estimation. The EM algorithm [37] is effective for handling latent variables. Therefore, it is adopted for parameter estimation.

### 3.2. Parameter Estimation

The fundamental principle of the EM algorithm is to start from initial estimates of the parameters of interest and alternately perform the E-step (Expectation) and the M-step (Maximization). In the E-step, the conditional expectation of the log-likelihood function is computed based on the observed data and current parameter estimates. In the M-step, this conditional expectation is maximized with respect to the target parameters. The parameter estimates are then updated, and the E and M steps are iterated until predefined convergence criteria are met. Given $\delta_{j}$, the likelihood function for the degradation increment of the $j^{th}$ sample under stress level $s_{i}$ is given by

(10) $$f\left(\Delta x_{i,j}|\delta_{j}\right)=\prod_{k=1}^{p_{ij}}\delta_{j}^{\frac{1}{2}}\frac{1}{\sqrt{2\pi \Delta \tau \left(t_{i,k}^{j},\gamma \right)}}\exp\left[-\frac{\delta_{j}{\left(\Delta x_{i,k}^{j}-a\exp\left(bs_{i}\right)\Delta \Lambda \left(t_{i,k}^{j},\theta \right)\right)}^{2}}{2\Delta \tau \left(t_{i,k}^{j},\gamma \right)}\right].$$

Based on the observed degradation data and the random effect $\delta_{j}$, the complete-data likelihood function is expressed as:

(11) LΘ=∏i=1m∏j=1nifΔxi,j|δjπδj|ν2,ν2=∏i=1m∏j=1ni∏k=1pij12πΔτti,kj,γδj12exp−δjΔxi,kj−aexpbsiΔΛti,kj,θ22Δτti,kj,γ∏i=1m∏j=1niν2ν2Γν2δje−ν2δjν2−1.

Taking the logarithm of both sides of Equation (11) yields the complete-data log-likelihood function.

(12) $$\ln L\left(\Theta \right)=\sum_{i=1}^{m}\sum_{j=1}^{n_{i}}\left[\frac{\nu }{2}\ln\frac{\nu }{2}-\ln\Gamma \left(\frac{\nu }{2}\right)+\left(\frac{\nu }{2}-1\right)\ln\left(\delta_{j}\right)-\frac{\nu }{2}\delta_{j}\right]+\sum_{i=1}^{m}\sum_{j=1}^{n_{i}}\left[-\frac{p_{ij}}{2}\ln\left(\delta_{j}\right)-\frac{p_{ij}}{2}\ln\left(2\pi \right)\right.\;-\frac{1}{2}\sum_{k=1}^{p_{ij}}\ln\left(\Delta \tau \left(t_{i,k}^{j},\gamma \right)\right)\left.-\sum_{k=1}^{p_{ij}}\frac{\delta_{j}{\left(\Delta x_{i,k}^{j}-a\exp\left(bs_{i}\right)\Delta \Lambda \left(t_{i,k}^{j},\theta \right)\right)}^{2}}{2\Delta \tau \left(t_{i,k}^{j},\gamma \right)}\right].$$

**E-step**: Let $\Delta x_{i,j}$ represent the degradation increment data for the $j^{th}$ product under stress level $s_{i}$, $\Theta =\left\{a,b,\theta ,\gamma ,v\right\}$, and ${\hat{\Theta }}_{e}=\left\{{\hat{a}}_{e},{\hat{b}}_{e},{\hat{\theta }}_{e},{\hat{\gamma }}_{e},{\hat{v}}_{e}\right\}$ denotes the parameter estimates obtained from the $e^{th}$ iteration. Given $\delta_{j}∼Ga\left(\frac{\nu }{2},\frac{\nu }{2}\right)$, the posterior distribution of $\delta_{j}|\Delta x_{i,j},\Theta$ is a Gamma distribution derived via Bayes’ theorem, i.e.,

(13) $$f\left(\delta_{j}|\Delta x_{i,j},\Theta \right)\infty \prod_{k=1}^{p_{ij}}\frac{1}{\sqrt{2\pi \Delta \tau \left(t_{i,k}^{j},\gamma \right)}}\delta_{j}^{\frac{1}{2}}\exp\left[-\frac{\delta_{j}{\left(\Delta x_{i,k}^{j}-a\exp\left(bs_{i}\right)\Delta \Lambda \left(t_{i,k}^{j},\theta \right)\right)}^{2}}{2\Delta \tau \left(t_{i,k}^{j},\gamma \right)}\right]\pi \left(\delta_{j}|\frac{\nu }{2},\frac{\nu }{2}\right)\;\infty \delta_{j}^{\frac{\nu +p_{ij}}{2}-1}\exp\left\{-\delta_{j}\left[\frac{\nu }{2}+\sum_{k=1}^{p_{ij}}\frac{{\left(\Delta x_{i,k}^{j}-a\exp\left(bs_{i}\right)\Delta \Lambda \left(t_{i,k}^{j},\theta \right)\right)}^{2}}{2\Delta \tau \left(t_{i,k}^{j},\gamma \right)}\right]\right\},$$

(14) $$\delta_{j}|\Delta x_{i,j},\Theta \sim Ga\left(\frac{\nu +p_{ij}}{2},\frac{\nu }{2}+\sum_{k=1}^{p_{ij}}\frac{{\left(\Delta x_{i,k}^{j}-a\exp\left(bs_{i}\right)\Delta \Lambda \left(t_{i,k}^{j},\theta \right)\right)}^{2}}{2\Delta \tau \left(t_{i,k}^{j},\gamma \right)}\right).$$

The conditional expectations of $\delta_{j}$ in the two states are obtained as follows:

(15) $$E_{1ij}\left(\Theta \right)=E\left(\delta_{j}|\Delta x_{i,j},\Theta \right)=\frac{\nu +p_{ij}}{\nu +\sum_{k=1}^{p_{ij}}\frac{{\left(\Delta x_{i,k}^{j}-a\exp\left(bs_{i}\right)\Delta \Lambda \left(t_{i,k}^{j},\theta \right)\right)}^{2}}{\Delta \tau \left(t_{i,k}^{j},\gamma \right)}},$$

(16) $$E_{2ij}\left(\Theta \right)=E\left(\ln\delta_{j}|\Delta x_{i,j},\Theta \right)=\psi \left(\frac{\nu +p_{ij}}{2}\right)-\ln\left[\frac{\nu }{2}+\sum_{k=1}^{p_{ij}}\frac{{\left(\Delta x_{i,k}^{j}-a\exp\left(bs_{i}\right)\Delta \Lambda \left(t_{i,k}^{j},\theta \right)\right)}^{2}}{2\Delta \tau \left(t_{i,k}^{j},\gamma \right)}\right],$$

where $\psi \left(\cdot \right)$ is the digamma function.

The expected value of the complete-data log-likelihood function is

(17) $$Q\left(\Theta |\Delta x_{1:m,1:n_{i}},{\hat{\Theta }}_{e}\right)=E\left[\ln L\left(\Theta \right)|\Delta x_{1:m,1:n_{i}},{\hat{\Theta }}_{e}\right]=\sum_{i=1}^{m}\sum_{j=1}^{n_{i}}\left[-\frac{p_{ij}}{2}E_{2ij}\left({\hat{\Theta }}_{e}\right)-\frac{p_{ij}}{2}\ln\left(2\pi \right)-\frac{1}{2}\sum_{k=1}^{p_{ij}}\ln\left(\Delta \tau \left(t_{i,k}^{j},\gamma \right)\right)\right.\;\left.-\sum_{k=1}^{p_{ij}}\frac{E_{1ij}\left({\hat{\Theta }}_{e}\right){\left(\Delta x_{i,k}^{j}-a\exp\left(bs_{i}\right)\Delta \Lambda \left(t_{i,k}^{j},\theta \right)\right)}^{2}}{2\Delta \tau \left(t_{i,k}^{j},\gamma \right)}\right]+\sum_{i=1}^{m}\sum_{j=1}^{n_{i}}\left[\frac{\nu }{2}\ln\frac{\nu }{2}-\ln\Gamma \left(\frac{\nu }{2}\right)+\left(\frac{\nu }{2}-1\right)E_{2ij}\left({\hat{\Theta }}_{e}\right)-\frac{\nu }{2}E_{1ij}\left({\hat{\Theta }}_{e}\right)\right]$$

**M-step:** ${\hat{\Theta }}_{\left(e+1\right)}=\arg\max Q\left(\Theta |\Delta x_{1:m,1:n_{i}},{\hat{\Theta }}_{e}\right)$, for Equation (17), partial derivatives with respect to parameters a, b, and ν are taken and set to zero. This yields that

(18) $${\hat{a}}_{e+1}=\frac{\sum_{i=1}^{m}\sum_{j=1}^{n_{i}}\sum_{k=1}^{p_{ij}}E_{1ij}\left({\hat{\Theta }}_{e}\right)\Delta x_{i,k}^{j}\exp\left(bs_{i}\right)\Delta \Lambda \left(t_{i,k}^{j},\theta \right)/\Delta \tau \left(t_{i,k}^{j},\gamma \right)}{\sum_{i=1}^{m}\sum_{j=1}^{n_{i}}\sum_{k=1}^{p_{ij}}E_{1ij}\left({\hat{\Theta }}_{e}\right)\exp\left(2bs_{i}\right)\Delta^{2}\Lambda \left(t_{i,k}^{j},\theta \right)/\Delta \tau \left(t_{i,k}^{j},\gamma \right)},$$

(19) $$\sum_{i=1}^{m}\sum_{j=1}^{n_{i}}\sum_{k=1}^{p_{ij}}{\hat{a}}_{e+1}\exp\left(bs_{i}\right)s_{i}E_{1ij}\left({\hat{\Theta }}_{e}\right)\Delta x_{i,k}^{j}\Delta \Lambda \left(t_{i,k}^{j},\theta \right)/\Delta \tau \left(t_{i,k}^{j},\gamma \right)-\;\sum_{i=1}^{m}\sum_{j=1}^{n_{i}}\sum_{k=1}^{p_{ij}}{\hat{a}}_{e+1}^{2}E_{1ij}\left({\hat{\Theta }}_{e}\right)\exp\left(2bs_{i}\right)s_{i}\Delta^{2}\Lambda \left(t_{i,k}^{j},\theta \right)/\Delta \tau \left(t_{i,k}^{j},\gamma \right)=0,$$

Substituting ${\hat{a}}_{e+1}$ into Equation (19) yields the updated estimate for b, denoted as ${\hat{b}}_{e+1}$.

(20) $$\sum_{i=1}^{m}\sum_{j=1}^{n_{i}}\left\{\ln\sum_{i=1}^{m}n_{i}-\ln\sum_{i=1}^{m}\sum_{j=1}^{n_{i}}E_{1ij}\left({\hat{\Theta }}_{e}\right)+\ln\frac{\nu }{2}-\psi \left(\frac{\nu }{2}\right)+E_{2ij}\left({\hat{\Theta }}_{e}\right)\right\}=0,$$

Solving Equation (20) yields the parameter ν, denoted by ${\hat{\nu }}_{e+1}$. Substituting Equations (18)–(20) into Equation (17) yields the profile log-likelihood function for θ and γ. The R function optim is employed to numerically obtain $\left({\hat{\theta }}_{e+1},{\hat{\gamma }}_{e+1}\right)$.

(21) $$\left(\hat{\theta },\hat{\gamma }\right)=\arg\;\max Q\left(\left(\hat{a},\hat{b},\theta ,\gamma ,\hat{v}\right)|\Delta x_{1:m,1:n_{i}},{\hat{\Theta }}_{e}\right).$$

**Figure 1:**
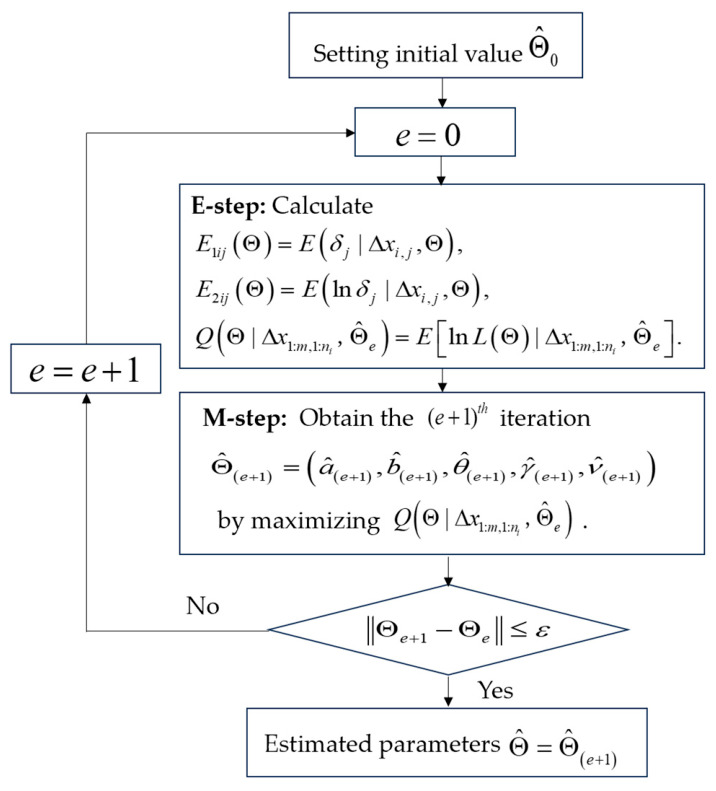
The EM algorithm flow chart.

Steps E and M are repeated until the difference between two consecutive iteration values is less than a specified threshold, $\left\|{\hat{\Theta }}_{\left(e+1\right)}-{\hat{\Theta }}_{e}\right\|<\varepsilon \left(\varepsilon =10^{-6}\right)$. Figure 1 depicts the flowchart of the EM algorithm. The estimates of $\left(b,\theta ,\gamma \right)$ are obtained by maximizing the profile log-likelihood function shown in Equation (7). This optimization is implemented using the MATLAB R2024a function ‘fminsearch’. The resulting parameter solutions are unique and exhibit stability across various initial values. Subsequently, the estimates ${\hat{\delta }}_{j}=1/{\hat{\sigma }}_{Bj}^{2}$ are derived by substituting the previously obtained values of $\left(b,\theta ,\gamma \right)$. Furthermore, these parameter estimates serve as the initial values ${\hat{\Theta }}_{0}$ for the EM algorithm.

## 4. Case Studies

### 4.1. Numerical Simulation

**Figure 2:**
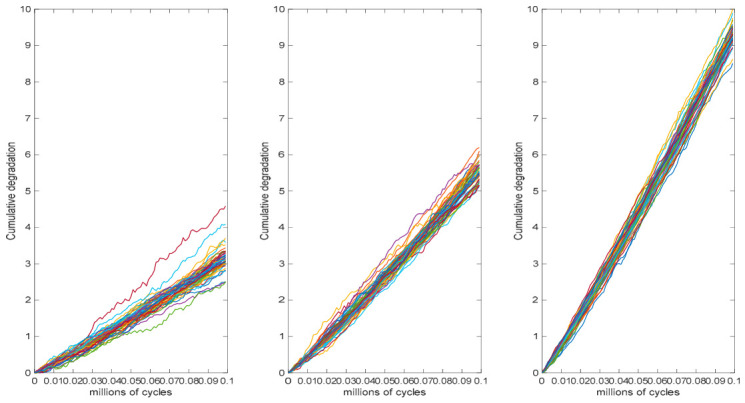
Crack growth accumulation at various stress levels.

Simulation data for fatigue crack propagation in an alloy product was generated. A constant-stress accelerated degradation model was employed to fit the simulation data and to estimate the failure lifetime and reliability function. Assuming that the accelerated stress for the alloy product is electrical stress, the accelerated stress levels are $S_{1}=1.15$ mA, $S_{2}=1.25$ mA, and $S_{3}=1.35$ mA; the normal stress level is $S_{0}=1$ mA, and the maximum stress level is $S_{m}=1.35$ mA. The relationship between the product drift coefficient and the accelerated stress level follows a power-law model, as defined by $\eta_{i}\left(b\right)=\exp(bs_{i})$, $s_{i}=\frac{\ln S_{i}-\ln S_{0}}{\ln S_{m}-\ln S_{0}}$, i=1,2,3. The normalized stress level is denoted by $s_{1}=0.4657$, $s_{2}=0.7436$, $s_{3}=1$. The sample size at each stress level is $n_{1}=n_{2}=n_{3}=50$, and each sample is measured at intervals of 0.001 million cycles, resulting in a total of 100 measurements. Under normal operating conditions, the product is considered to have failed once the crack length reaches the threshold of D=5 inches. The true parameter values are set to a=16, ν=5, b=2. $\tau \left(t;\theta \right)=t^{\theta }$, θ=1.1 and $\tau \left(t;\gamma \right)=t^{\gamma }$, γ=1.3 which are two monotonically non-decreasing functions of time t. Repeat the simulation step 200 times. Figure 2 illustrates the corresponding crack degradation trajectories under three accelerated stress levels.

Using simulation data from CSADT on an alloy product, the effectiveness of the proposed method is demonstrated. As shown in Figure 2, the crack degradation data exhibit pronounced nonlinear characteristics, necessitating the use of a nonlinear model to describe the degradation process. Since the accelerated stress is electrical, a power-law model is selected as the acceleration model. The drift coefficient is modeled as a function of stress, while a random effect is introduced into the diffusion coefficient to account for unit-to-unit variability. The model based on a generalized nonlinear Wiener process that accounts for random effects in the diffusion coefficient is denoted as $M_{1}$. The nonlinear Wiener process proposed by Si et al. [13], in which the drift coefficient depends on the accelerated stress level and the diffusion coefficient incorporates a random effect, is denoted as $M_{2}$. The accelerated degradation model without random effects, in which all parameters of the generalized nonlinear Wiener process are fixed, is denoted as $M_{3}$. The means and standard deviations (SD) of the parameters, derived from the EM algorithm as detailed in Section 3.2, are listed in Table 1.

The Akaike Information Criterion (AIC) [38] is an information-theoretic criterion used to evaluate model fit. Proposed by Akaike based on information theory, it is now widely applied in model selection for degradation modeling. To compare the fitting performance of different models, the AIC is adopted as the selection criterion. Based on the AIC principle, a smaller value indicates a superior fit to the degradation data. The AIC is defined as $AIC=2p-2\ln L\left(\Theta \right)$, where p denotes the number of parameters in the parameter set Θ, and $\ln L\left(\Theta \right)$ represents the maximum value of the likelihood function. $BIC=p\ln(n)-2\ln L\left(\Theta \right)$, n is the sample size [39]. Based on the above evaluation criterion, model $M_{1}$ yields the smallest AIC and BIC values, and its estimated parameters are closer to the true values. This indicates that model $M_{1}$ provides the best overall fit and is more suitable for reliability analysis of this type of product. Given the complexity of the degradation process, the accelerated degradation model incorporating diffusion heterogeneity is better suited for the observed data. Under normal stress levels, the estimated mean time to failure (MTTF), $MTTF_{j}=E\left(T|S_{0}\right)=$$\int_{0}^{\infty }tf_{j}\left(t|S_{0}\right)dt$, j=1,2,3 and the corresponding 95% confidence intervals (CIs) are summarized in Table 2. The true MTTF is 0.4074 million cycles. Model $M_{1}$ yields an MTTF estimate of 0.4118, which is closer to the true value. In contrast, models $M_{2}$ and $M_{3}$ slightly overestimate the product’s MTTF.

**Figure 3:**
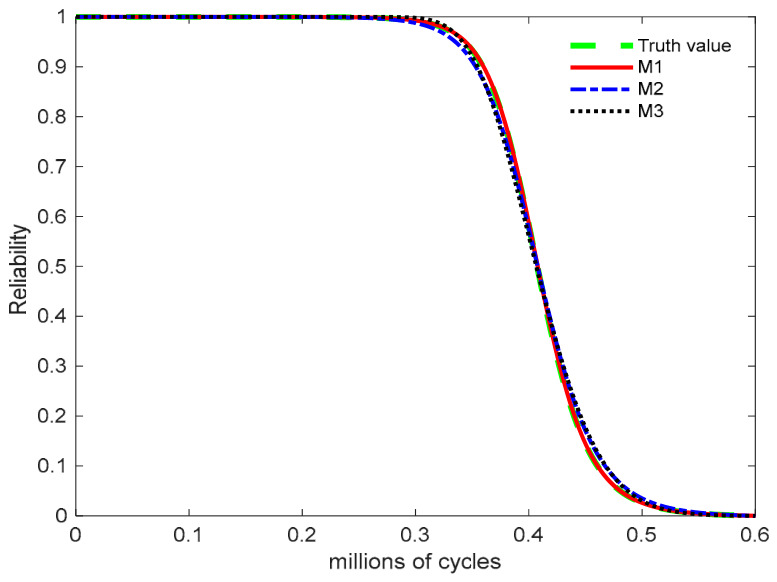
Reliability curves of metal failure lifetime distribution under normal stress.

By substituting the parameters from Table 2 into Equations (5) and (6), the PDFs and reliability function of the failure lifetime distribution for the alloy product under the normal stress level are obtained. Figure 3 presents the reliability curves for the true values and models $M_{1}$, $M_{2}$, and $M_{3}$ under the normal stress level of $S_{0}=1$ mA. The reliability curves indicate that the degradation process transitions from gradual deterioration to rapid failure. The inflection point of this transition corresponds to the steepest segment of the reliability curve. From a physical perspective, this point represents the critical time at which the product shifts from gradual degradation to accelerated failure, marking the interval where the failure rate increases sharply and failure probabilities become densely concentrated. The key criterion for evaluating model quality lies in its ability to accurately capture both the location of the inflection point and the slope of the reliability decline. Model $M_{1}$ closely aligns with the true curve regarding both the inflection point and the decline rate, exhibiting the highest agreement with the true reliability curve. In contrast, models $M_{2}$ and $M_{3}$ slightly overestimate the true reliability.

**Figure 4:**
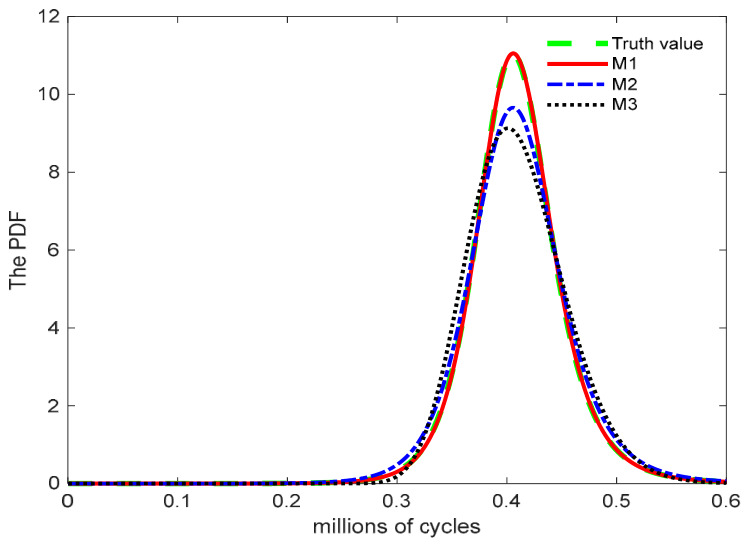
Probability density curves of the failure lifetime distribution of metal under normal stress.

Figure 4 presents the PDF curves corresponding to the true values and models $M_{1}$, $M_{2}$, and $M_{3}$. A comparison of the failure lifetime PDFs indicates that model $M_{1}$ aligns well with the true distribution, reasonably capturing both the central tendency and dispersion. In contrast, the failure lifetime distributions of models $M_{2}$ and $M_{3}$ exhibit more pronounced right-skewness than the true distribution. The 95% confidence interval for the failure lifetime estimated by model $M_{1}$ is narrower than those of models $M_{2}$ and $M_{3}$, indicating superior predictive accuracy.

**Figure 5:**
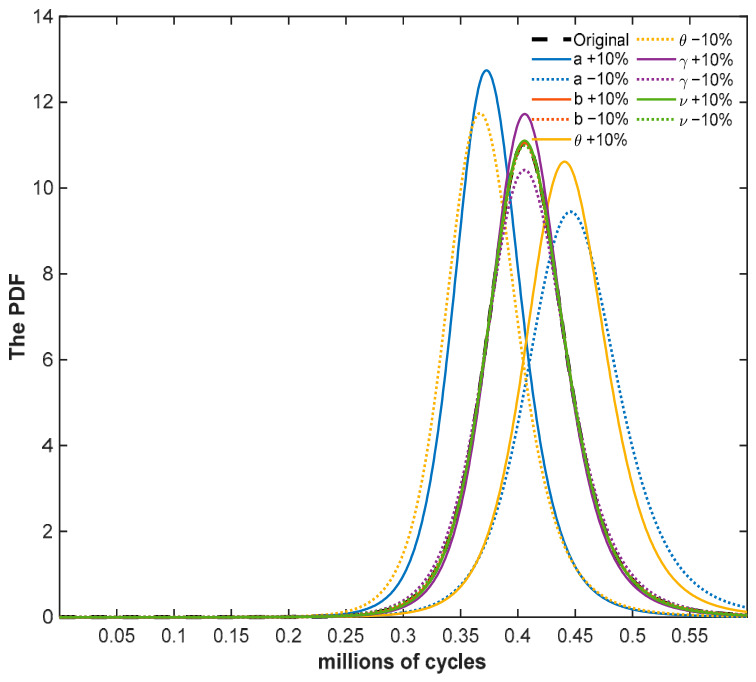
Sensitivity analysis of $M_{1}$ with a fluctuation range of ±10%.

To analyze the sensitivity of the MTTF to variations in each parameter, this paper conducts a sensitivity analysis on the key parameters $\Theta =\left\{a,b,\theta ,\gamma ,\nu \right\}$. Based on model $M_{1}$, a deviation of ±10% is introduced to the estimated parameter values. Figure 5 illustrates the sensitivity analysis [40] results for the PDF of failure lifetime. The results indicate that variations in parameters a and θ have the most significant impact on the accuracy of failure lifetime prediction; in contrast, variations in parameters b,γ, and ν have a relatively minor effect. Therefore, priority should be given to the accuracy of parameters a and θ during the model solution process.

### 4.2. Application of Metal Wear Width Data

**Figure 6:**
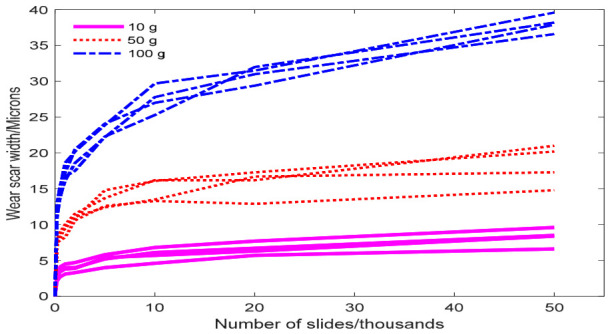
Degradation trajectories of metal wear width data at accelerated stress levels.

The constant-stress accelerated test data reported in Table C.19 of the literature [17] were used to validate the proposed methodology. To thoroughly investigate the wear mechanism of a specific alloy, sliding wear tests were conducted under different applied loads to obtain wear scar width data (μm). An exponential model was adopted as the acceleration model, with stress levels set at 10 g, 50 g, and 100 g. Four samples were tested at each stress level, and their degradation responses were recorded at multiple time points. The normal use stress level was 5 g, the maximum stress level was 100 g, and the corresponding normalized stress levels were $s_{1}=0.0526$, $s_{2}=0.4737$, and $s_{3}=1$. Under the normal use stress level, failure was defined as the wear width reaching 10 μm. Figure 6 illustrates the degradation trajectories of the wear width measured in sliding wear tests under three stress levels, exhibiting pronounced nonlinear characteristics. The volatility of the degradation trajectories varies significantly across stress levels. The results of Bartlett’s test indicate that p=0.2736, at a significance level of α=0.05; the null hypothesis of equal variances across groups cannot be rejected, suggesting that the degree of data fluctuation is essentially consistent. Therefore, it is necessary to incorporate volatility heterogeneity into the degradation model.

Since the accelerated stress in accelerated degradation testing is a load-related quantity, an exponential model is selected as the acceleration model. An accelerated degradation model, denoted as $M_{1}$, is developed based on a generalized nonlinear Wiener process, where the drift coefficient is modeled as a function of stress and the diffusion coefficient incorporates random effects. We also consider a nonlinear Wiener process model [13] where the drift coefficient depends on the accelerated stress level and the diffusion coefficient incorporates a random effect; this model is denoted as $M_{2}$. In addition, a generalized nonlinear Wiener process accelerated degradation model without random effects, where all model parameters are fixed, is denoted as $M_{3}$. The parameters of models $M_{1}$, $M_{2}$, and $M_{3}$ were estimated using the EM algorithm, with the results reported in Table 3. The number of bootstrapping is 1000.

The AIC and BIC were employed to evaluate the goodness of fit of each model. As shown in Table 4, model $M_{1}$ exhibits the highest log-likelihood value and the lowest AIC and BIC values among the models. This result indicates that model $M_{1}$ provides the best overall fit and is more suitable for characterizing the sliding wear degradation process of the alloy. Given the complexity of product degradation processes, the accelerated degradation model $M_{1}$, which incorporates diffusion coefficient heterogeneity, demonstrates substantially superior fitting performance compared with model $M_{3}$, which ignores random effects. Therefore, volatility heterogeneity should be explicitly considered in degradation modeling. Under normal stress conditions, the MTTF and the corresponding 95% CIs for each model are reported in Table 4. As indicated in Table 4, the mean number of sliding cycles to failure estimated by model $M_{1}$ is $7.8185\times 10^{4}$ cycles.

**Figure 7:**
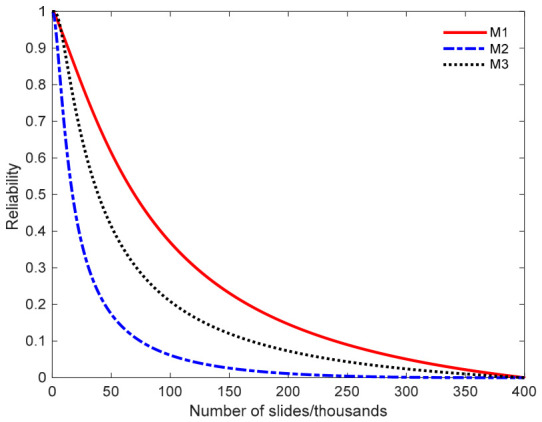
Reliability curves of product failure lifetime under different models.

**Figure 8:**
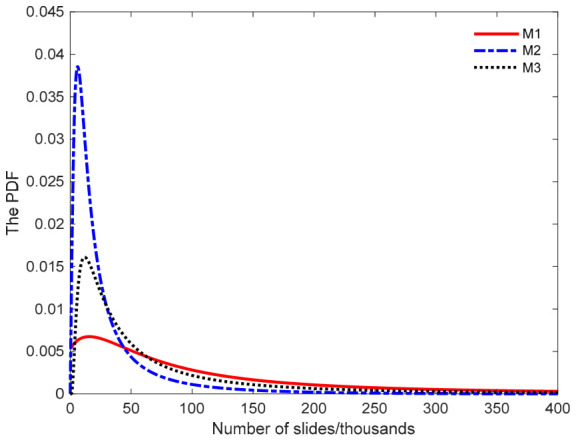
Probability density curves of product failure lifetime under different models.

Substituting the parameters from Table 3 into Equations (5) and (6) yields the PDFs and reliability function of the metal failure lifetime under normal stress levels. As shown in Figure 7, reliability predictions vary substantially across modeling approaches. Using model $M_{2}$ for reliability analysis may lead to premature assessments because its reliability curve declines prematurely, overestimating the wear rate. Such predictions yield overly conservative assessments of failure lifetime, prompting premature maintenance and unnecessary resource waste. In practical engineering, these alloy products often retain significant remaining service life, and premature replacement would incur unnecessary costs. Similarly, the reliability curve for $M_{3}$ also declines more rapidly than that of $M_{1}$, leading to an underestimation of the predicted reliability. Using $M_{3}$ for reliability prediction similarly results in premature maintenance, thereby increasing operational costs through unnecessary service interventions. In summary, employing either model $M_{2}$ or $M_{3}$ for reliability prediction leads to premature assessments of alloy product lifespan. This negatively affects production planning and resource optimization, increasing operational costs. By contrast, model $M_{1}$ provides more accurate predictions, better balancing wear effects and production efficiency, and thus aligning more closely with engineering practice. Figure 8 presents the PDF curves for the $M_{1}$, $M_{2}$, and $M_{3}$ models. The PDFs of product failure lifetime derived from the three models all exhibit left-skewness. Among them, the mean failure time estimated by the $M_{1}$ model aligns more closely with the observed degradation patterns, while models $M_{2}$ and $M_{3}$ yield markedly different predictions. This further demonstrates that the proposed model, which accounts for diffusion coefficient heterogeneity, provides superior evaluation performance and more objectively reflects the true distribution of product failure lifetime.

## 5. Conclusions

In this study, an accelerated degradation model was developed by linking the drift coefficient to the stress level. The model accounts for heterogeneity in diffusion coefficients—capturing the effects of internal or external factors—by incorporating a Gamma-distributed random effect. The EM algorithm was employed for parameter estimation, enabling subsequent model comparison and reliability analysis. The effectiveness of the proposed method was demonstrated using crack propagation data and metal wear scar width data from an alloy product. Comparative results indicate that the model accounting for diffusion coefficient heterogeneity achieves higher estimation accuracy than models neglecting such heterogeneity. The proposed generalized Wiener process model proved to be more effective in characterizing such degradation processes. Several avenues warrant further investigation in future research. For instance, this study focuses on heterogeneity in the diffusion coefficient, whereas the drift coefficient may also exhibit stochasticity. Future work could explore models that simultaneously account for heterogeneity in both the drift and diffusion coefficients. Additionally, while this research focuses on single-stress models, multi-stress scenarios warrant further in-depth investigation.

## Figures and Tables

**Table 1:** Simulation degradation data parameter estimation results.

	a	b	θ	γ	ν	$\sigma_{B}^{2}$
Truth value	16	2	1.1	1.3	5	-
$M_{1}$ SD	15.9851 0.2702	2.0014 0.0175	1.1001 0.0025	1.3071 0.0088	5.1105 0.5947	- -
$M_{2}$ SD	15.9847 0.2807	2.0012 0.0029	1.0999 0.6734	1 -	5.6076 0.0174	- -
$M_{3}$ SD	16.0059 0.3351	1.9999 0.0208	1.1000 0.0031	1.3052 0.0138	-	1.6521 0.1610

**Table 2:** Model evaluation results of simulated degradation data.

	$\ln L\left(\Theta \right)$	AIC	BIC	MTTF	CI
Truth value	-	-		0.4074	[0.3274, 0.5013]
$M_{1}$	34,516	−69,022	−69,007	0.4079	[0.3285, 0.5012]
$M_{2}$	34,063	−68,118	−68,106	0.4083	[0.3176, 0.5106]
$M_{3}$	32,659	−65,308	−65,293	0.4088	[0.3306, 0.5046]

**Table 3:** Model Parameters for Metal Wear Width Data.

	a	b	θ	γ	ν	$\sigma_{B}^{2}$
$M_{1}$ SD	3.6456 0.3186	1.5582 0.0926	0.2014 0.0033	0.3368 0.0368	3.1603 0.7675	- -
$M_{2}$ SD	3.7481 0.3643	1.5071 0.1072	0.2048 0.0079	1 -	3.5318 1.5898	- -
$M_{3}$ SD	4.3593 0.2054	1.3745 0.0516	0.1992 0.0034	0.3993 0.0365	- -	1.6180 0.2298

**Table 4:** Model Evaluation Results for Metal Wear Width Data.

	$\ln L\left(\Theta \right)$	AIC	BIC	MTTF (Thousands)	CI (Thousands)
$M_{1}$	−115.4586	240.9172	243.3417	78.1851	[4.3276, 343.8023]
$M_{2}$	−157.8913	323.7826	325.7222	24.4529	[2.0580, 152.0671]
$M_{3}$	−125.3561	260.7122	260.6518	59.9996	[3.0029, 282.4801]

## Data Availability

Data are contained within the article.

## Abbreviations

The following abbreviations are used in this manuscript:

ADT	Accelerated degradation test
CSADT	Constant-stress accelerated degradation test
SSADT	Step-stress accelerated degradation test
MLE	Maximum likelihood estimation
EM	Expectation–maximization
FPT	First passage time
PDF	Probability density function
AIC	Akaike information criterion
MTTF	Mean time to failure
CI	Confidence interval
